# Começa a Temporada de Frio nos Trópicos. A Crioablação da Fibrilação Atrial no Brasil

**DOI:** 10.36660/abc.20200658

**Published:** 2020-09-18

**Authors:** Nilson Araújo de Oliveira

**Affiliations:** 1 Instituto D’Or de Ensino e Pesquisa Rio de Janeiro RJ Brasil Instituto D’Or de Ensino e Pesquisa (IDOR),Rio de Janeiro, RJ - Brasil

**Keywords:** Fibrilação Atrial, Veias Pulmonares, Crioablação, Congelamento, Cateteres Cardíacos

O isolamento das veias pulmonares é a pedra angular da ablação para fibrilação atrial. O início dos episódios de fibrilação pelo disparo rápido de taquicardias atriais que se originam dessas estruturas está bem documentado. A eliminação desses gatilhos pelo isolamento elétrico do antro da veia pulmonar está associada a um melhor controle da arritmia e menos eventos adversos, tanto nas formas paroxística e persistente de fibrilação atrial. Entretanto, este não é o único mecanismo envolvido, especialmente nas formas mais avançadas. Um número enorme de diferentes técnicas de ablação foi descrito para abordar esses outros mecanismos. Apesar dos resultados promissores iniciais, nenhuma outra abordagem mostrou eficácia em grandes estudos randomizados multicêntricos até agora.^1^

A obtenção de um isolamento duradouro das veias pulmonares não é uma tarefa simples. Os cateteres de ablação padrão foram projetados para eliminar uma pequena área circunscrita do tecido cardíaco, o que é desejável para circuitos de arritmia como o Wolff-Parkinson White, mas quando se deseja abordar grandes áreas de tecido em estruturas tridimensionalmente complexas, como é o antro das veias pulmonares, esta tarefa se torna um desafio. O desenvolvimento da navegação não-fluoroscópica, pontas irrigadas e a medida em tempo real da força de contato, entre outras melhoras notáveis dos cateteres, foram cruciais para a adoção da ablação da fibrilação atrial como procedimento de rotina na eletrofisiologia clínica. Entretanto, a ablação com esses cateteres ainda é realizada por formação de lesões pontuais contíguas..

A crioablação é uma técnica antiga para tratamento de tumores, entre outras doenças.^2^ As sondas cirúrgicas evoluíram para criocateteres para tratamento invasivo de arritmias cardíacas há muitos anos. Embora a radiofrequência tenha se tornado o método padrão para ablação cardíaca, a crioablação apresenta algumas vantagens, como a possibilidade de utilizar grandes áreas para a criação de lesões teciduais. Balões que congelam todo o tecido cardíaco do antro da veia pulmonar foram desenvolvidos e o isolamento da veia pulmonar com um única ação tornou-se realidade. A desvantagem desses dispositivos é a incapacidade de tratar lesões fora desta região específica.

Foram realizadas comparações entre radiofrequência e crioablação para tratamento de fibrilação atrial em ensaios randomizados multicêntricos e essas técnicas parecem iguais em termos de taxa de sucesso e segurança.^3^ Esses ensaios são realizados geralmente em centros com bastante experiência, com um alto número de procedimentos complexos de ablação. Extrapolar os resultados de qualquer ensaio para centros com baixo volume de procedimentos é preocupante. É sabido que as taxas de sucesso e complicação da ablação por fibrilação atrial têm uma correlação inversa com o número de procedimentos realizados por ano em um centro de ablação. A crioablação parece necessitar de um número menor de procedimentos para obter bons resultados e segurança quando comparada à radiofrequência.^4^

O Brasil é um país grande, com muitas diferenças regionais. A maioria da população depende do sistema público de saúde (SUS) para ter acesso a tratamentos médicos. A análise cuidadosa de novas modalidades de tratamento justifica-se para comprovar seus reais benefícios na realidade do sistema de saúde brasileiro. Uma publicação recente demonstrou reduções marcantes de custo para o sistema de saúde em pacientes submetidos à ablação de fibrilação atrial no Brasil,^5^ o que parece reforçar a importância da ablação no manejo de pacientes com fibrilação atrial em nosso país. Outro estudo demonstra de maneira muito elegante o custo-benefício da crioablação em comparação com a ablação por radiofrequência no sistema público de saúde brasileiro.^6^

Neste artigo, Boghossian et al.^7^ descreveram resultados iniciais notáveis com crioablação em um centro brasileiro, com baixo índice de complicações quando comparado a um registro nacional.^8^ Os tempos de fluoroscopia foram comparáveis aos de outras publicações, mas apresentam uma clara desvantagem quando tempos de fluoroscopia extremamente baixos ou mesmo nulos são obtidos hoje em dia com o mapeamento eletroanatômico. Embora não mencionadas pelos autores, as belas imagens geradas pelo ecocardiograma transesofágico do balão criogênico anexado ao antro pulmonar neste trabalho sugerem a possibilidade de uma redução adicional da exposição à fluoroscopia à medida que a experiência com o método se desenvolva. A ocorrência de paralisia do nervo frênico, uma temida complicação da crioablação, também foi comparável à da literatura e a maioria dos casos foi autolimitada. Outro achado interessante são os bons resultados obtidos nos estágios mais avançados da doença, sugerindo que uma abordagem mais conservadora possa ser considerada no tratamento inicial desses pacientes.

No futuro, os dispositivos de ablação de ação única desempenharão um papel crescente no tratamento de pacientes com fibrilação atrial. Esperamos que esses avanços tecnológicos levem à possibilidade de tratar cada vez mais pacientes com melhores resultados, segurança e custo-efetividade.
