# Supplementary material for: Começa a Temporada de Frio nos Trópicos. A Crioablação da Fibrilação Atrial no Brasil
Source: Arq Bras Cardiol. 2020 Sep 18;115(3):536–7. [Article in Portuguese] doi: 10.36660/abc.20200658 (PMC9363095; doi:10.36660/abc.20200658)
Supplement: Translation

# Translation: The Cold Season Begins in the Tropics. Cryoablation for Atrial Fibrillation in Brazil

**Authors:** Nilson Araújo de Oliveira

**Affiliations:** 1 Brazil Instituto D’Or de Ensino e Pesquisa (IDOR), Rio de Janeiro, RJ - Brazil

**Keywords:** Atrial Fibrillation, Pulmonary Veins, Cryoablation, Freezing, Cardiac Catheters

Pulmonary vein isolation is the cornerstone for atrial fibrillation ablation. The onset of fibrillation episodes by rapid firing of atrial tachycardias originating from these structures is well documented. The elimination of these triggers by electrical isolation of the pulmonary vein antrum is associated with better arrhythmia control and fewer adverse events, in both the paroxysmal and persistent forms of atrial fibrillation. However, this is not the only mechanism involved, especially in more advanced forms of this arrhythmia. An enormous amount of different ablation techniques has been described to address these other mechanisms. Despite the initial promising results, no other approach has shown effectiveness in large multicenter randomized trials until now.^1^

Obtaining durable pulmonary vein isolation is not a simple task. The standard ablation catheters were designed to ablate a small circumscribed area of cardiac tissue, which is desirable for arrhythmia circuits like Wolff-Parkinson White, but it is a challenge when it is necessary to ablate large areas of complex tridimensional structures, such as the pulmonary vein antrum. The development of non-fluoroscopic navigation, irrigated tips and contact force real-time measurement, among other catheter remarkable improvements, were crucial for the adoption of atrial fibrillation ablation as a routine procedure in clinical electrophysiology. However, ablation with these catheters are still performed by point-by-point lesion deployment.

Cryoablation is an old technique for the treatment of tumors, among other diseases.^2^ Surgical cryoprobes evolved to cryocatheters for the invasive treatment of cardiac arrhythmias many years ago. Although radiofrequency ended up becoming the standard method for cardiac ablation, cryoablation has some advantages, such as the possibility of using large surface areas for the ablation probe. Balloons that freeze the entire pulmonary vein antrum cardiac tissue were developed and the “single-shot” pulmonary vein isolation became a reality. The disadvantage of these devices is the inability to treat lesions outside the pulmonary vein antrum.

Comparison of radiofrequency and cryoablation for atrial fibrillation treatment in multicenter randomized trials were performed and these techniques seem equal in terms of success and safety.^3^ These trials are typically performed in well-experienced centers, with a high number of complex ablation procedures. Extrapolating the outcomes of any complex ablation trial to low-volume centers is a matter of concern. It is well known that the success and complication rates of atrial fibrillation ablation have an inverse correlation to the number of procedures performed by year in an ablation center. Cryoablation seems to require a lower number of procedures to perform them with good results and safety when compared to radiofrequency.^4^

Brazil is a large country, with many regional differences. The majority of the population depends on the universal public health system (SUS, *Sistema Único de Saúde*) to have access to medical treatments. A careful analysis of new treatment modalities is justified to demonstrate their real benefits within the reality of the Brazilian health system. A recent publication, demonstrated marked cost reductions for the healthcare system in patients submitted to atrial fibrillation ablation in Brazil,^5^ which seems to reinforce the importance of ablation for the management of patients with atrial fibrillation in our country. Another study very elegantly demonstrated the cost benefits of cryoablation when compared to radiofrequency ablation in the Brazilian public health system.^6^

In this paper, Boghossian et al.^7^ described remarkable initial results with cryoablation in a Brazilian center, with a low complication index when compared to a national registry.^8^ The fluoroscopy times were comparable to other publications but are a clear disadvantage when extremely low or even zero fluoroscopy times are obtained nowadays with electroanatomical mapping. Although not mentioned by the authors, the beautiful images generated by transesophageal echocardiography of the cryoballoon attached to the pulmonary antrum in this paper suggest the possibility of further reduction in fluoroscopy exposure as the experience with the method develops. The occurrence of phrenic nerve palsy, a feared complication of cryoablation, was also comparable to that in the literature and most of cases were self-limited. Another interesting finding is the good results obtained at more advanced stages of the disease, suggesting that a more conservative approach may be considered for the initial treatment of these patients.

In the future, “single-shot” ablation devices will play an increasing role in the management of atrial fibrillation patients. We expect that these technological advances will lead to the possibility of treating an increasing number of patients with better results, safety and cost-effectiveness.
